# Monitoring tobacco use among a sample of Tunisian high school pupils

**DOI:** 10.1093/eurpub/ckac131.570

**Published:** 2022-10-25

**Authors:** N Zammit, A Amara, R Ghammam, S Ben Fredj, S Boujebha, A Maatouk, M Ouertani, W Ben Belgacem, J Maatoug, H Ghannem

**Affiliations:** 1 Epidemiology, University Hospital Farhat Hached, Sousse, Tunisia; 2 Faculty of Medicine of Sousse, University of Sousse, Sousse, Tunisia; 3 LR19SP03, University Hospital Farhat Hached, Sousse, Tunisia; 4 Department of Family and Community Medicine, Faculty of Medicne of Sousse, University of Sousse, Sousse, Tunisia; 5 LR12ES03, Faculty of Medicne of Sousse, University of Sousse, Sousse, Tunisia

## Abstract

**Background:**

Smoking is the leading cause of preventable death. This risk behavior usually begins during adolescence. However, only the third of the countries are monitoring this risk behavior among adolescents.

**Objective:**

To assess the prevalence of tobacco use and to determine the predictors of its experimentation among high school students from Sousse (Tunisia) in 2018 and 2019.

**Methods:**

In 2018 and 2019, two cross-sectional studies were led among 1399 and 1342 adolescents randomly selected from the same four high schools in Sousse. For data collection, the same pre-tested questionnaire was self-administered anonymously to the participants in the presence of pre-trained investigators.

**Results:**

Girls represented 60.5% and 63.2% of participants in 2018 and 2019 respectively. The prevalence of tobacco experimentation was of 29.4% in 2018 and of 26.7% in 2019. Current cigarette smoking was objectified in 9.8% and 7.4% of participants in 2018 and 2019. Regardless of the year of the study, the main predictors of lifetime tobacco use among them were: current use of e-cigarette (adjusted OR of 6.4 [4.5-9.0]), cannabis experimentation (adjusted OR of 5.3 [2.7-10.7] and alcohol consumption (adjusted OR of 3.9 [2.5-6.3]).

**Conclusions:**

Experimentation and current use of tobacco are common among the high school students of Sousse. The national smoking prevention program should be reinforced by multisectoral prevention actions targeting not only tobacco use but also the consumption of other substances.

**Key messages:**

• Tobacco experimentation is high among the adolescents of Sousse.

• Tobacco experimentation is strongly associated with other substances use among the adolescents of Sousse.

